# Cardiac output monitoring using the LiDCOplus™ monitor in abdominal aortic surgery: changes in calibration factor in aortic aneurysm disease versus aortic occlusive disease

**DOI:** 10.1186/cc10830

**Published:** 2012-03-20

**Authors:** HK Jørgensen, J Bisgaard, T Gilsaa

**Affiliations:** 1 Lillebaelt Hospital, Kolding, Denmark

## Introduction

Monitoring of cardiac output (with subsequent haemodynamic optimisation) may improve outcome after high-risk surgery. The pulmonary artery catheter is still considered the gold standard, but has potential serious complications. Much effort has been put into developing equally good, but less invasive techniques. One of these, the LiDCOplus™ system, uses pulse power analysis to calculate cardiac output and is calibrated by a lithium indicator dilution technique. Since cardiac output is affected by the compliance of the aorta, the LiDCO calculates a calibration factor (CF) each time it is calibrated. The purpose of this study was to investigate whether insertion of aortic prosthetic material would affect aortic compliance and thereby the CF. It was hypothesised that the change in CF would be larger in patients with aortic occlusive disease (AOD) than in patients with aortic aneurysm disease (AAD), since previous studies have shown that these two groups differ considerably on both haemodynamic capacity and their response to aortic cross-clamping [1].

## Methods

A prospective study in 51 patients undergoing open elective abdominal aortic surgery - 30 patients with AAD and 21 with AOD. CF values were obtained at baseline, before induction of anaesthesia (T1) and 30 minutes after reperfusion (T2).

## Results

AAD patients were older (70 vs. 65 years, *P *< 0.05), predominantly males (80% vs. 47%), weighed more (80 kg vs. 73 kg, *P *< 0.1) and preoperative cardiac co-morbidity was more prevalent (43% vs. 14%). No difference was found in the use of epidural analgesia, vasopressors, or inotropes between the groups. At T1, CF was significantly higher for AAD = 0.83 versus AOD = 0.68 (*P *= 0.01). After reperfusion, T2, there was no significant difference in CF, AAD = 0.86 versus AOD = 0.81 (*P *= 0.53). The percentage change in CF from T1 to T2 was significantly larger in AOD than in AAD (20% vs. 1.3%) (*P *< 0.05).

## Conclusion

Operative insertion of an abdominal aortic prosthesis significantly affects the calibration factor in patients with AOD, indicating an increase in aortic compliance and the need for recalibration of the LiDCOplus™. No significant change was seen in patients with aortic aneurysm disease.
